# Improving the quality of GP referrals to the Croydon Assessment & Liaison Team

**DOI:** 10.1192/bjo.2021.548

**Published:** 2021-06-18

**Authors:** Gabriella Lewis, Lucia Chaplin, Gareth Knott, Alexandra Coull, Lamide Sobamowo

**Affiliations:** South London and Maudsley NHS Foundation Trust

## Abstract

**Aims:**

To increase the percentage of GP referrals to the Croydon Assessment & Liaison (A&L) Team deemed to be of ‘good quality’. The A&L Team receives a large number of referrals daily from GPs, and it was identified that many of these referrals did not include important and relevant information, leading to delays in patient assessments.

**Method:**

A questionnaire was distributed to A&L MDT members to collect information about what information they consider important in a GP referral. The project team reviewed the results of the questionnaire, along with current policies and guidelines, to create a set of criteria by which to assess the quality of GP referrals, as there was no pre-existing gold standard available. A random sample of 6 GP referrals per week stratified by locality was collected and assessed against these criteria.

Using Plan-Do-Study-Act (PDSA) methodology change ideas were generated, and a GP referral form was identified as an important intervention to adopt. A previously-developed draft form was updated after a round of consultations with various stakeholders including Assessment & Liaison staff, GPs and the CCG. The new GP referral form was uploaded to the GP DSX electronic referrals platform and GP practices were also emailed directly to encourage them to use the new form.

The proportion of GP referrals deemed to be of good quality was compared pre and post-intervention. Uptake of the new GP referral form was recorded as a process measure, and the length of time taken to discuss referrals at A&L daily referrals meetings as a counterbalance measure.

**Result:**

At baseline 33% of GP referrals were deemed to be of good quality using the developed criteria. This improved to 58% after implementation of the new referral form in January 2021. There was poor overall uptake of the form, with only 32.5% of GP referrals utilising the new form so far, however of the referrals received on the new form 69% fulfilled the criteria for good quality. Comparison of length of discussion required for referrals with and without the new form showed no significant difference (7.7 and 7.6 minutes respectively).

**Conclusion:**

Implementation of a standardised GP referral form was effective at increasing the proportion of referrals deemed to be of good quality. However, further PDSA cycles focused on improving uptake of the form will be required.

